# Effect of Fluoride on Cytotoxicity Involved in Mitochondrial Dysfunction: A Review of Mechanism

**DOI:** 10.3389/fvets.2022.850771

**Published:** 2022-04-19

**Authors:** Mingbang Wei, Yourong Ye, Muhammad Muddassir Ali, Yangzom Chamba, Jia Tang, Peng Shang

**Affiliations:** ^1^College of Animal Science, Tibet Agriculture and Animal Husbandry College, Linzhi, China; ^2^The Provincial and Ministerial Co-founded Collaborative Innovation Center for R&D in Tibet Characteristic Agricultural and Animal Husbandry Resources, Linzhi, China; ^3^Institute of Biochemistry and Biotechnology, University of Veterinary and Animal Sciences, Lahore, Pakistan

**Keywords:** fluoride, apoptosis, mitochondrial dysfunction, cytotoxicity, ROS

## Abstract

Fluoride is commonly found in the soil and water environment and may act as chronic poison. A large amount of fluoride deposition causes serious harm to the ecological environment and human health. Mitochondrial dysfunction is a shared feature of fluorosis, and numerous studies reported this phenomenon in different model systems. More and more evidence shows that the functions of mitochondria play an extremely influential role in the organs and tissues after fluorosis. Fluoride invades into cells and mainly damages mitochondria, resulting in decreased activity of mitochondrial related enzymes, weakening of protein expression, damage of respiratory chain, excessive fission, disturbance of fusion, disorder of calcium regulation, resulting in the decrease of intracellular ATP and the accumulation of Reactive oxygen species. At the same time, the decrease of mitochondrial membrane potential leads to the release of Cyt c, causing a series of caspase cascade reactions and resulting in apoptosis. This article mainly reviews the mechanism of cytotoxicity related to mitochondrial dysfunction after fluorosis. A series of mitochondrial dysfunction caused by fluorosis, such as mitochondrial dynamics, mitochondrial Reactive oxygen species, mitochondrial fission, mitochondrial respiratory chain, mitochondrial autophagy apoptosis, mitochondrial fusion disturbance, mitochondrial calcium regulation are emphasized, and the mechanism of the effect of fluoride on cytotoxicity related to mitochondrial dysfunction are further explored.

## Introduction

Fluoride is abundantly existing in our environment in different forms and it can cause serious harm to the ecological environment and human health. Worldwide numerous cases of fluorosis have been observed. Especially, in China, the fluorine pollution in soil is of serious concern, as it is causing a greater negative impact on human health and ecological environment (1). The situation of fluorosis in 20 countries showed that about 260 million people suffer from fluorosis caused by various fluorine sources (2). In 1997, fluorosis in China showed that about 31 million people showed signs of fluorosis (3), including about 21 million dental fluorosis patients and 10 million skeletal fluorosis patients (DDCMH, China). A large amount of fluoride is deposited into the environment, which in the long run will lead to excessive fluorine content in the soil and water environment, which ultimately penetrate into agricultural products, and resulting in excessive fluorine content in crops (4). A survey is shown that most of the fluorine pollution in China comes from iron and steel, phosphate fertilizer and electrolytic aluminum, in which the iron and steel industry is the biggest cause of fluorine pollution (5). He et al. (6) analyzed the fluorine pollution in 122 kinds of vegetables and 36 topsoil samples within 10 kilometers range of the aluminum plant abandoned for 5 years. It was observed that the fluorine content of 89.26% of the agricultural products was beyond maximum permissible level of food pollutants in China.

Fluoride is a necessary element in the normal development and growth of animals, but excessive fluoride will cause damage to the animal body. A large number of phenomena showed that the typical manifestations of fluorosis are; dental fluorosis (7), hypertension (8), skeletal fluorosis (9), reproductive disorders (10) and dementia (11). A large amount of fluoride deposition mainly damages to bones, teeth, and central nervous system via passing through the blood-brain barrier that can lead to insomnia or somnolence and other symptoms (12). There is evidence that excessive fluoride intake can damage various organs including brain (13, 14), liver (15, 16), kidney (17), ovary (18). Fluoride in the environment into the body is mainly absorbed by the stomach and intestines, through the cell membrane, deposited in bone and soft tissue, causing damage to bone and soft tissue. The most important deposition point is in the liver, and the excretion point is mainly the kidney (19). Long-term exposure to fluoride lead to the accumulation of fluoride in liver tissue, affect the activity of antioxidant enzymes and the expression of cyclooxygenase, seriously destroy the ultrastructure of hepatocytes, cause significant pathological changes of liver tissue, and destroy the balance of the body (20). Higher concentration of sodium fluoride increases the expression of phosphorus (P), potassium (K), blood urea nitrogen (BUN), uric acid (UA) and creatinine (CRE), and induce renal histopathological damage and lead to apoptosis (21). It also increased the mRNA and protein expression levels of death receptor (FAS), tumor necrosis factor (TNF), TNF-related apoptosis-inducing ligand (TRAIL), Caspase 8, Caspase 3 and poly (ADP-ribose polymerase) on the cell surface. It has been reported that long-term drinking water with high levels of fluoride has a higher risk of developing dementia than drinking water with relatively low levels of fluoride (6). Moreover, long-term exposure to fluoride can significantly increase the expression of mRNA related to the neurotoxicity of fluoride and down-regulate the pathways related to learning and memory in the hippocampus of their offspring, indicating that long-term exposure to fluoride lead to brain nervous system damage (22). Neurotoxicity caused by fluoride lead to apoptosis, production of reactive oxygen species, impaired mitochondrial dynamics and decreased ability of antioxidant enzymes (23–25). A large number of studies have shown that absorbed fluoride can damage the structure and function of tissues and cells, such as thyroid (26), spleen, cecal tonsilla lymphocytes, human neuroblastoma SH-SY5Y cells, ameloblasts and Leydig cells, resulting in apoptosis (27, 28). At the same time, in recent years, apoptosis induced by fluoride through mitochondria has attracted wide attention.

It is well known that high concentrations of fluoride can lead to a certain degree of toxicity in organs or systems, such as primer oxidative stress, cell cycle arrest and apoptosis, resulting in nuclear condensation, nuclear membrane rupture, mitochondrial vacuolation, fragmentation and mitochondrial fission (as shown in Table 1). Fluoride causes cytotoxicity by interfering with the mechanism of enzyme action and calcium metabolism (55). Certain amount (1–2%) of fluoride can cause mucosal inflammation and necrosis (56). In fluoride induced neuronal damage, inhibition of mitochondrial fission is involved in autophagy and excessive apoptosis (57). The cellular damage mechanism of fluoride is usually via protein inhibition, organelle destruction, pH change and electrolyte imbalance (58, 59). Absorption of elevated concentrations of fluoride can lead to strong DNA damage, mainly due to DNA single-strand or double-strand DNA breakage (60) caused by oxidative stress of free radicals. In fewer cases of fluoride-amino acid binding, it is shown that most of the metal protein active sites in related proteins bind to fluoride or fluoride-metal complexes which act as substrate mimics (58, 61). Fluoride inhibits nutrient transport, cell respiration and glycolysis (62, 63) through the inhibition of metalloproteins (64). At the cellular level, long-term exposure to fluoride lead to electrolyte imbalance due to inhibition of transmembrane proteins, mitochondrial abnormalities, metabolic disruption and stress signal induction (65–67). Long-term exposure to fluoride causes the permanent damage of mitochondria and abnormal respiratory chain, mainly due to the reduction of ATP produced by anaerobic glycolysis (ATP → ADP+AMP+H^+^), the release of protons, leading to intracellular acidification, leading to oxidative stress (62, 64, 68). Moreover, fluoride irreversibly destroys cells and organelles via destroying the unfolded protein response pathway of endoparasitic reticulum (69, 70), directly removing Ca^2+^ and ER proteins or stimulating oxidative stress signals, and (71, 72) disturbing Ca^2+^ balance (73) in mitochondria via excessive release of Ca^2+^. The activation of G protein in the Golgi apparatus (74) by fluoride leads to Ca^2+^-dependent activation of exocytosis. Long-term exposure of fluoride irreversibly damages the integrity of the mitochondrial membrane (75), resulting in mitochondrial dysfunction. Fluoride can change mitochondrial permeability, consume ATP, inhibit the normal operation of cellular respiratory chain, reduce mitochondrial activity, and release cytochrome C, resulting in oxidative stress (76, 77).

**Table 1:** Apoptosis mechanism related to mitochondrial disturbance caused by fluorosis.

**Pathways of apoptosis**	**Mechanism**	**Related factors**	**References**
Energy metabolic pathway	It leads to the inhibition of the expression / activity of respiratory chain complex, the insufficient production of ATP, the accumulation of ROS in cells, and the apoptosis of cells.	NDUFV2, SDHA, CYC1	(29, 30)
	It leads to the decrease of the activity of key enzymes in mitochondrial intima and the production of ATP, which leads to cell apoptosis.	ATP5J, ATP5H, The ATP synthase	(31–34)
Reactive oxygen species pathway	Destroy the oxygen homeostasis, so as to destroy the normal biological process, from the mitochondrial permeability transition pore (mPTP) opening mechanism, increase mmp, resulting in the content of CtyC, caspase 3, caspase 8, caspase 9, RARP, the release of ROS oxidative stress signal, resulting in the increase of ROS and apoptosis.	mmp, Cty C, caspase 3, caspase 8, caspase 9, RARP	(35, 36)
	ROS interacts with purine bases, pyrimidine bases and ribose to increase the content of messenger RNA, destroy single or double strands of DNA, activate DNA-dependent proteases and p53, and cause cell apoptosis.	DNA dependent protease, P53	(37)
	It causes a small amount of electrons to escape from the electron chain, forms peroxides, aggravates oxidative stress, causes mitochondrial complex I to produce ROS and activates NLRP3, ASC and caspase 1.	NLRP3, ASC, caspase 1	(38)
	Cells stop in G0/G1 phase, which aggravates apoptosis.	/	(35)
	The intermembrane gap protein is released after permeating into the outer membrane of the mitochondria, activating cystatin and leading to apoptosis.	Smac, Diabio, Endonuclease G	(39)
Autophagy and apoptosis	Inhibit cell proliferation, induce apoptosis and autophagy. After fluoride invasion, autophagy apoptosis pathway was activated, and apoptosis factor, caspase, ATG protein and p53 regulated autophagy.	BCL-2, Bax, caspase, ATG protein, P53	(40)
	Fluoride invades as long as it attacks the main autophagy pathway of mitochondria	Sirt1/FoxO3a, PINK1/Parkin, Nix/ BNIP3L, BNIP3 and FUNDC1, PHB2	(41–45)
	Cause autophagy to accumulate in small volume and induce autophagy injury and inhibit the release of Cyt C.	Cyt C	(46)
Fission and fusion	The synergistic action of fission molecules leads to the breaking of the balance of fission and fusion and the morphological changes of mitochondria.	Drp1, Dyn2	(47, 48)
	Decrease the level of fission protein and increase the level of fusion protein	Cyt C, caspase 3, caspase 9, Mfn1, Mfn2, Fis1, Pro-caspase9	(49, 50)
Calcium pathway	F invasion can combine with Ca^2+^ to form insoluble CaF_2_ and reduce F absorption and cytotoxicity.	/	(51, 52)
	Ca^2+^ strictly controls the entry of ATP and ROS, F^−^ into cells in a simple way. Mitochondria absorb Ca^2+^, through mitochondrial Ca^2+^ unidirectional receptors and then flow into mitochondria through Ca^2+^ unidirectional transporters (MEU), manipulating energy metabolism, resulting in ROS accumulation and cell apoptosis.	MEU	(53)
	ER- mitochondria-calcium-apoptosis: caspase activation leads to the increase of Ca^2+^ content, which stimulates the release of CytC and pro-apoptotic factors into mitochondria. CytC and APAF-1 form apoptotic bodies, which indirectly act on the upstream signal of apoptosis, leading to downstream caspase 3 and caspase 7 division, resulting in cell apoptosis.	Caspase 3, caspase 7, Cyt C, BcL-2, Bax	(54)

Mitochondria are the main energy-supplying organelles of cells, which are closely related to the regulation of physiological function of eukaryotic cells (78, 79). Mitochondria play an important part in energy metabolism, apoptosis, cell differentiation, cell signal transduction and iron metabolism (10, 80, 81). After long-term exposure to fluoride or absorption of abnormal concentrations of fluoride, the structure of mitochondria in cells and tissues is seriously damaged. This results in decrease of number of mitochondria, abnormal activity of the respiratory chain, increase of oxidative stress, gene damage and biogenic dysfunction. Fluoride can induce the balance between fission and fusion of mitochondria, resulting in abnormal function and morphology of mitochondria. Fluoride induced mitochondrial kinetic damage and oxidative stress in SH-SY5Y cells revealed that sodium fluoride (NaF) could increase the level of fusion protein and significantly reduce the level of protein fission in rat hippocampus, indicating that fluoride leads to the occurrence of human Alzheimer's disease (10) by inhibiting mitochondrial fission, autophagy and excessive apoptosis. Studies have demonstrated that fluoride enters the body, resulting in the production and accumulation of a large amount of ROS in the brain, an increase in lipid and nucleic acid oxidation, and a decrease in the activity of antioxidant enzymes. Zhou et al. also found that fluoride exposure can lead to mitochondrial damage and ROS accumulation (82–85) in mouse lymphocytes, which further indicates that fluorosis can lead to mitochondrial fission / fusion imbalance and increase of intracellular ROS.

## Potential Molecular Mechanism of Mitochondrial Dysfunction Involved in Cell Damage Induced by Fluorosis

### Molecular Mechanisms of Energy Metabolism Involved in Fluoride-Induced Cell Injury

Numerous study states that excessive intake of fluoride lead to cell dysplasia or decrease the ability of cell proliferation and differentiation. The main reason is that, excessive fluoride destroys the ultrastructure of cell mitochondria and interfere with the expression of respiratory chain complex, being expected to result in a decrease in the content of ATP and an increase in the content of ROS. Some studies have shown that excessive fluoride intake cause mitochondrial dysfunction and reduce the production of ATP in cardiomyopathy (15), hepatocytes (84), kidney cells (86), granuloma cells (85), oocytes (87). Mitochondrial respiratory chain is the principal link of ATP production, and ATP production depends on the mitochondrial respiratory chain complex (88) in the mitochondrial inner membrane. Once the transmission of mitochondrial respiratory chain is blocked, ATP synthesis will be affected, resulting in mitochondrial dysfunction. Mitochondrial respiratory chain complex I (NDUFV2), mitochondrial respiratory chain complex II (SDHA) and mitochondrial respiratory chain complex III (CYC1) play an important role in regulating mitochondrial function and are the key links between ATP production (29, 30). Mitochondrial respiratory chain complex I (NDUFV2) and mitochondrial respiratory chain complex II (SDHA) are the main receptors for electron entry into the mitochondrial electron transfer chain (30, 89, 90), which manage the entry and exit of related ions inside and outside the membrane; mitochondrial respiratory chain complex III (CYC1) is called the gatekeeper of mitochondrial respiratory chain, which maintains the normal production capacity of mitochondria. Mitochondrial respiratory chain complex I (NDUFV2) mainly catalyzes NADH oxidation, while mitochondrial respiratory chain complex II (SDHA) mainly catalyzes succinic acid oxidation to fuming acid (91). Mitochondrial respiratory chain complex III (including CYC1) is an important target of mitochondrial oxidative phosphorylation and the third main source of reactive oxygen species (92). Once the expression or activity of the mitochondrial respiratory chain complex is blocked, it can lead to insufficient production of ATP and accumulation of ROS in mitochondria, resulting in severe mitochondrial dysfunction. Abnormal expression of mitochondrial respiratory chain complex can cause mitochondrial dysfunction. Mitochondrial respiratory chain complexes such as NDUFV2, SDHA and CYC1 are involved in intracellular oxidative phosphorylation and ATP synthesis. The expression of NDUFV2, SDHA and CYC1 in heart increased significantly after excessive fluoride treatment, which may be related to the damage of the mitochondrial respiratory chain. At the same time, change of mitochondrial membrane will affect the transmission of mitochondrial respiratory chain. ATP5J and ATP5H are key enzymes in mitochondrial intima, which can promote the process of oxidative phosphorylation to increase the synthesis of ATP. Ovarian granulose cells were severely damaged after exposure to fluoride, and the content of ATP in the ovary was significantly reduced. The mechanism of this phenomena was mainly due to the damage of its sub cellular organelles (mitochondria, ribosomes, endoparasitic reticulum, geology apparatus, etc.). The mitochondria showed vacuolation, crest fracture, dissolution, etc. It was assumed that mitochondria were destroyed when granulose cells were exposed to fluoride during the development and maturation of follicles and oocytes, resulting in insufficient cell energy supply and massive cell damage. These results further suggest that the normal expression of the mitochondrial respiratory chain complex and ATP5J and ATP5H play a crucial role in the maintenance of mitochondrial function in ovarian granulose cells (93). It has been reported that the content of ATP in liver and kidney decreased after fluoride treatment, and the expression of important ATP synthase ATP5J and ATP5H decreased significantly, which further indicated that fluoride or fluoride could destroy the main synthase of the mitochondrial respiratory chain. Previous studies have shown that fluoride has negative effects on sperm morphology (94), captivation (95), overestimation (96), acrosome reaction and fertilization ability (97, 98). Mammalian sperm need a lot of energy to complete fertilization in the process of energy acquisition and hyperventilation, while glycolysis and mitochondrial respiration are the key links of energy generation (99). Mitochondrial respiration is considered to be the main source of ATP production, and the production efficiency of ATP is higher than that of glycolysis (100, 101), indicating that fluorine's harm to sperm mainly lies in reducing sperm ATP production and vitality. The study reported that in fluorosis areas, the incidence of heart disease is associated with long-term exposure to fluoride; such heart disease symptoms are often characterized by atherosclerosis, myocardial infarction and high blood pressure. The regular contraction and relaxation of the heart are mainly caused by the production of ATP in myocardial fibers and mitochondria of cardiomyopathy. The content of total phosphorus decreased after fluoride-induced cardiomyopathy injury. Mitochondrial ATP synthase plays an important role in the synthesis of ATP in mitochondria, which is mainly composed of two parts: water-soluble protein complex F_1_ and hydrophobic part F_0_. ATP5J and ATP5H were closely related to ATP synthesis. After fluoride stimulation, the mRNA expression and protein levels of ATP5J and ATP5H increased significantly, but ATP production decreased. Therefore, the increased expression of ATP5J and ATP5H compensates for the mitochondrial dysfunction induced by fluoride, which further lead to the decrease of ATP synthesis. It has been reported that mitochondrial respiratory chain damage can induce a sharp increase of intracellular ROS.

### Potential Molecular Mechanisms of Fluoride-Induced Mitochondrial ROS Involved in Cell Damage Induced by Fluorosis

Fluorine is a small molecular element with active polarity and often exists in nature. Some studies have shown that long-term exposure to fluoride and exceeding the threshold of the body will not only cause damage to various tissues of the body (102), but also invade the central nervous system and produce dementia (103). Some studies have also shown that fluoride can lead to fetal mental retardation, mainly because fluoride can break through the placental barrier, enter the fetal brain, and accumulate in the fetal brain (104). Some studies have shown that ROS-mediated dysfunction of mitochondrial respiratory chain can lead to fluoride-induced cell damage (105). Some studies have shown that fluoride may transform the apoptosis pathway into necrosis, which shows the increase of inhibitory apoptosis factors. Mitochondria can cause cell injury or apoptosis by producing ROS, pro-inflammatory signals or through mitochondrial membrane permeability (106). Excessive ROS can damage the structure and function of mitochondria, and when mitochondrial dysfunction occurs, the oxygen homeostasis in the tissue is destroyed, resulting in the damage of normal cellular biological processes. It is released from mitochondria through the mitochondrial permeability conversion pore (mPTP) opening mechanism (107). When ROS is used as a signal of oxidative stress, the increase of intracellular reactive oxygen species (ROS) can activate the signal pathway of apoptosis. Once the apoptosis stimulus signal is present, excessive ROS production contributes to macromolecular oxidation, leading to free radicals attacking membrane phospholipids, leading to membrane damage by inducing lipid peroxidation, mitochondrial membrane depolarization, and apoptosis (38). Mitochondria is both ROS targets and sources of additional ROS production. Studies have demonstrated that the release of cytochrome C (CytC) may lead to the production of ROS (108). At the same time, previous studies have shown that the formation of reactive oxygen species opens the mitochondrial permeability conversion pore injection (mPTP drug) and increases the destruction of mitochondrial membrane potential (MMP), which then causes the subsequent increase in Cyt C, leading to the reduction of Caspase-3 and eventual apoptosis (36). Studies have shown that ROS can interact with the purine base, pyrimidine base and ribose. At the same time, ROS can destroy a single or double strand of DNA and activate DNA-dependent protein kinases and P53, leading to cell apoptosis (37). Studies confirmed that NaF could improve HL-60 cells (109), testicular interstitial cells (110), H9C2 cardiomyopathy (111), oocytes (112), human lung BeAS-2B cells (113), rat liver and thymocytes (114), CytC, caspase-3, caspase-9 mid or protein levels are associated with apoptosis. After exposure to fluoride, the expression of caspase-3, caspase-9 and caspase-8 in fish kidney and porcine hepatocytes increased in a dose-dependent manner, indicating that fluoride can induce Caspase-dependent apoptosis. In addition, Buckalew detected activated caspase-8, caspase-3, and polyhidrosis diphosphate ribose polymerase (PARP) by western blotting and found induction of apoptosis in ameloblasts (35). Secondly, Apoptosis caused by mitochondrial disorders is caused by oxidative damage (39), and the increase of ROS in cells can make cells stay in the G0/G1 phase, thus aggravating cell death (35). The release of cytomembrane gap proteins such as Smac/Diablo and endonuclease G after permeating the outer membrane of mitochondria can promote cell apoptosis by activating cysteine. Mitochondria continuously reduce oxygen by adding electrons resulting in the formation of many ROS and RNS, including superoxide, hydrogen peroxide, hydroxyl radical, hypochlorous acid, peroxynitrite anion and nitric oxide, which aggravate the oxidative stress of cells and lead to further apoptosis (39). A large number of studies in cell culture and experimental animal models have shown that the accumulation of fluoride in the brain leads to the production of more ROS, increased oxidation of lipids and nucleic acids, and decreased activity of antioxidant enzymes (115). ROS is a by-product of metabolism. A small number of electrons may escape from the mitochondrial electron transport chain, resulting in the production of superoxides. Increased oxidant load also promoted the production of additional ROS from mitochondrial complex I to further enhance cell oxidative stress and promote cell death (117). In addition, ROS produced by mitochondria can activate NLRP3, adaptive proteins ASC and caspase-1 to form inflammatory bodies, and the accumulation of damaged mitochondria aggravates inflammation and leads to cell damage. The change of mitochondrial permeability is another important cause of cell death, which leads to the dissipation of mitochondrial transmembrane potential and the cessation of oxidase (116). In addition, it can lead to rapid apoptosis necrosis (116). When apoptosis was induced by mitochondrial damage, the ATP produced by mitochondria through the respiratory chain decreased or ROS increased (118). Complexes I, II, III and IV from respiratory chains with ubiquinones and Cytc, and their inhibition can reduce the electron transport kinetics and Cytc.

### Fluorine-Induced Mitochondrial Ca^2+^ Regulations Are a Potential Molecular Mechanism Involved in Fluoride-Induced Cell Damage

**Figure 1:**
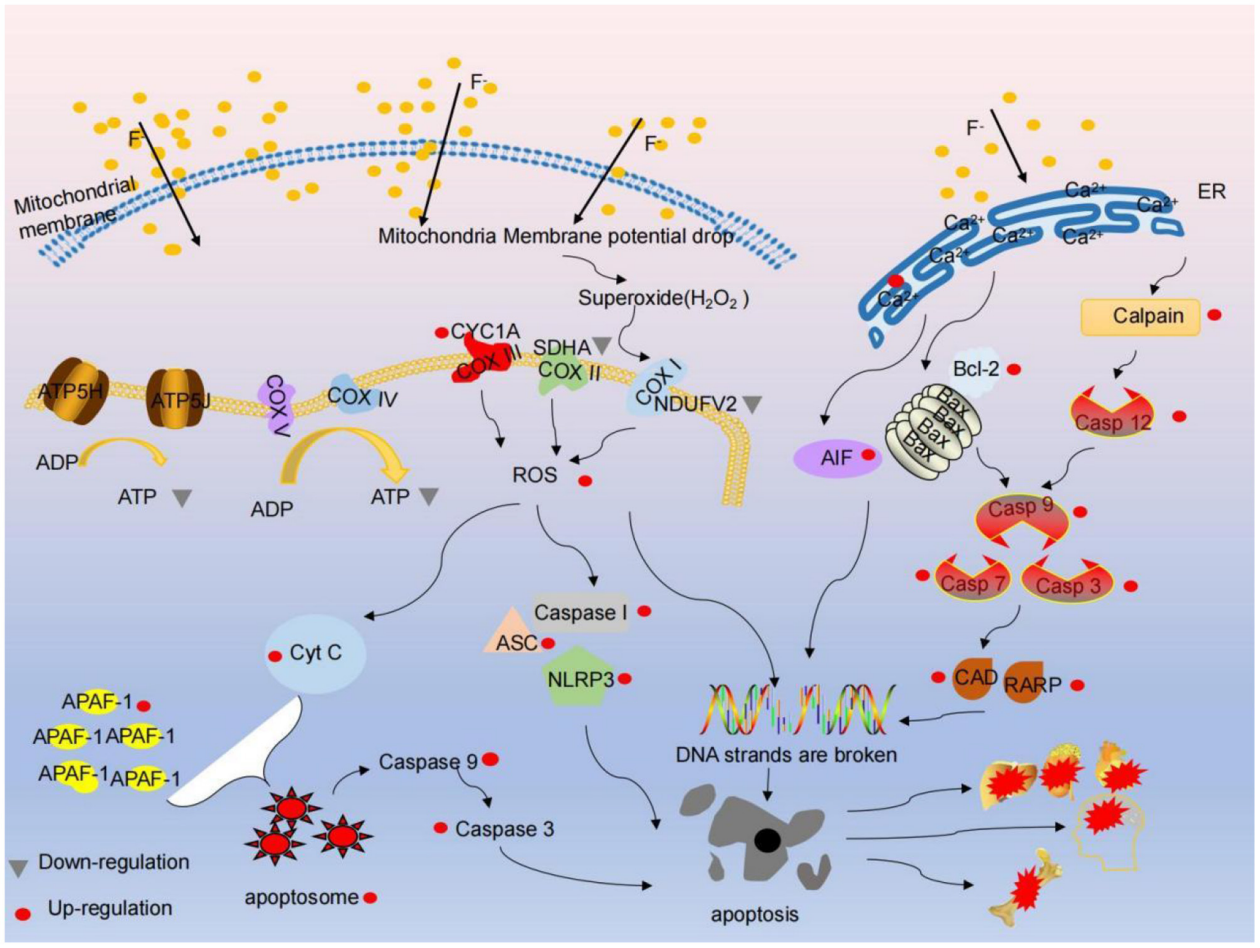
Fluoride cause energy metabolism, mitochondrial respiratory chain damage, ER- mitochondrial-calcium-apoptotic pathway, resulting in apoptosis, resulting in dementia, skeletal fluorosis, hepatomegaly toxicity and other symptoms. (1) F^−^ entered the mitochondria by free diffusion, the activities of key enzymes of respiratory chain complexes COX I, COX II, Cox III and ATP decreased, protein expression levels of NDUFV2 and SDHA decreased, and ROS increased. At the same time, the decrease of ATP synthesis and the decrease of mitochondrial membrane potential caused the release of Cytc, which further triggered the caspase cascade reaction, which gave rise to cell apoptosis. (2) F^−^ enters the mitochondria by free diffusion, and a small amount of electrons escape from the respiratory chain to form peroxides (H_2_O_2_), which act on COX I, produce extra ROS, activate NLRP3, ASC and Caspase 1, and cause apoptosis. (3) A large amount of ROS can destroy the single strand / double strand of DNA and is expected to result in apoptosis. (4) F-activates Ca^2+^ dependent enzyme captain and apoptosis factor Bcl-2 through ER stress, which further triggers caspase cascade reaction, releases CAD and RARP, DNA single strand / double strand destruction, and further causes cell apoptosis. It eventually causes symptoms such as dementia, skeletal fluorosis, hepatomegaly toxicity.

The latest evidence shows that fluoride can lead to soft tissue damage, and the degree of fluoride injury depends on the concentration of fluoride, exposure time and organ type (28). Fluorine has a pronounced effect on the bones of the body. When fluorine is absorbed by the blood, it is rapidly distributed all over the body and finally mainly accumulates in calcium-rich tissues such as bones and teeth. Excessive fluoride will make the collagen fibers of tibia loose, curved and heterogeneous, widen the bone lacuna space, decrease bone plasticity, reduce bone tolerance and increase the probability of fracture; excessive fluoride mainly leads to bone damage. Recently, it has been noted that the right amount of calcium can relieve fluorosis (52). A large number of literature have shown that F and Ca have a strong affinity. Supplementary Ca forms a novel complex, insoluble CaF_2_, in the intestine by combining with F, which reduces the absorption and toxicity of fluoride (51). However, a large amount of serum calcium into bone tissue will lead to increasing bone mineral density, hyperosteogeny, hypocalcemia. The proliferation rate of osteoblasts reduced and apoptosis increased in fluorosis. Mitochondrial pathway is part of the most important pathways of apoptosis. Mitochondria are the main energy centers and the main source of ATP and ROS. Their functions are strictly controlled by Ca^2+^. In the process of fluoride poisoning, mitochondrial Ca^2+^ uptake is needed in order to meet energy supply and demand, while maintaining reduced antioxidant capacity to prevent excessive release of ROS (119). Some studies have concluded that the content of fluoride and the level of Ca^2+^ in cells exposed to fluoride increased. This may be the product of simple diffusion of fluoride into the cell (120). The accumulation of fluoride may increase the release of excess Ca^2+^ from intracellular Ca^2+^ reserve, which makes cells vulnerable to damage. On the other hand, TSCE treatment decreased the fluoride content and Ca^2+^ level of fluoride-exposed cells. Mitochondria absorb Ca^2+^ through mitochondrial Ca^2+^ unidirectional receptor. Mitochondrial calcium unipolar (MCU) is the main mediator of Ca flowing into mitochondria, manipulating cell energy metabolism. ROS production and programmed cell death, all of which are essential for fluorosis (53). Studies have shown that fluoride may induce osteoblast apoptosis and mitochondrial dysfunction by increasing endoparasitic reticulum stress, and then activate the endoparasitic reticulum pathway induced by calcium. Fluoride induces endoparasitic reticulum stress; however, long-term endoparasitic reticulum stress induces apoptosis (121). Ca^2+^ is involved in almost all physiological activities and is the main messenger of the endoplasmic reticulum. Endoplasmic reticulum calcium pool is the main calcium pool in osteoblasts, and endoplasmic reticulum stress can induce increased intracellular Ca^2+^ level. Ca^2+^ activates Ca^2+^ dependent enzymes, such as Calpain, and divides and activates Caspase 12. Once activated, Caspase 12 acts at promoters (Caspase 9) and effectors (Caspase 7 and Caspase 3) to induce apoptosis (54). In particular, recent studies have demonstrated the relationship between ER, Mitochondria and calcium-apoptotic connection. In mitochondrial-mediated Caspase activation, the increase in intracellular Ca^2+^ level is effectively transmitted to mitochondria by pro-apoptotic BCL-2 family proteins (Bax and Bak), and indirectly acts as an important upstream signal of apoptosis. The increase of mitochondrial calcium level stimulated the release of pro-apoptotic molecules such as CtyC. In addition, CtyC and APAF-1 formed apoptosis bodies and treated pro-Caspase 9, which led to the division of downstream Caspase 3 and Caspase 7 in the cytoplasm and endoplasmic lumen (as shown in Figure 1).

### Fluoride-Induced Mitochondrial Fission and Fusion Are Involved in the Potential Molecular Mechanism of Cell Damage Induced by Fluorosis

**Figure 2:**
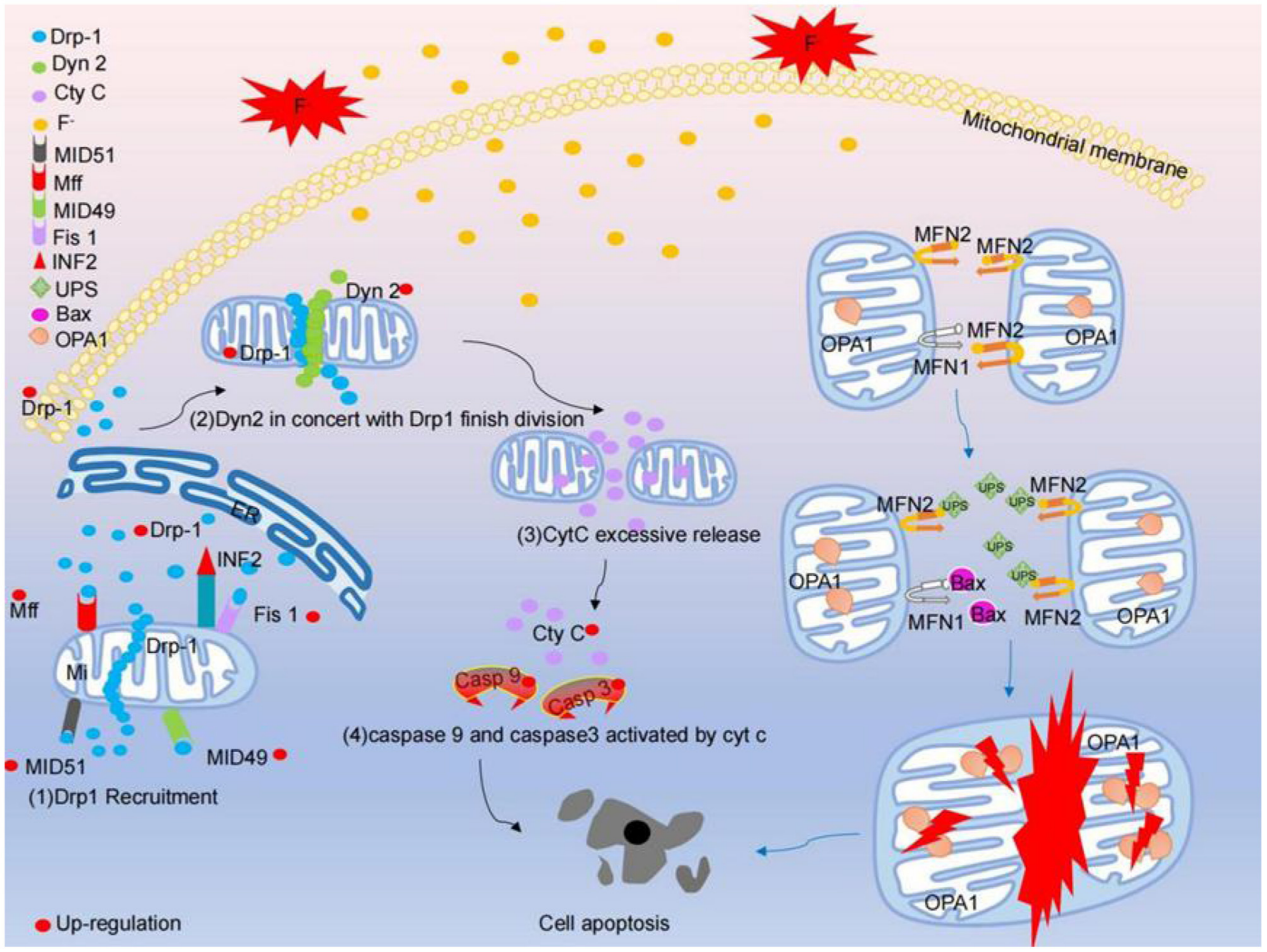
Fluoride stimulates mitochondria, causing excessive mitosis and fusion disorders that result in apoptosis. Fluoride stimulates the fission pathway of mitochondria: (1) The expression of Mff, Fis1, MiD49 and MiD51 on the surface of mitochondria increased, and Drp1 was recruited from cytoplasm and endoplasmic reticulum. A large number of Drp1 form a circular structure in the middle of the mitochondria and compress the mitochondria. (2) Dyn2 and Drp1 are up-regulated together to finalize the division of mitochondria. (3) Excessive mitosis of mitochondria lead to structural damage of mitochondria, resulting in excessive release of Cyt c from the interior of mitochondria in the cytoplasm. (4) Caspase 9 and Caspase 3 are activated and eventually lead to apoptosis (5) (50). The mitochondrial fusion pathway stimulated by fluoride: after fluoride stimulation, the binding of mitochondrial surface fusion protein MFN1 to Bax increased, the UPS (ubiquitin-proteasome) in mitochondrial surface fusion protein MFN2 degraded, and the activity of mitochondrial membrane fusion protein OPA1 decreased, which comprehensively destroyed the process of mitochondrial fusion and finally led to apoptosis.

Mitochondria play an important role in apoptosis, cell signal transduction and iron metabolism, which are related to mitochondrial dynamics, which are the process in which mitochondria form a network through the dynamic balance of fission and fusion. Fluorosis can cause mitochondrial dysfunction, which is partly due to the disruption of the balance between mitochondrial fission and fusion, which leads to changes in the morphology of mitochondria, which in turn leads to mitochondrial dysfunction (122). The neurotoxicity of fluoride is related to the destruction of mitochondria (123). Mitochondrial fission / fusion kinetics is very important for maintaining functional mitochondria (124). MID49, MID51, Mff, Fis-1, Drp1 and Dyn2 are necessary fission molecules to maintain mitochondrial division. However, the fusion between the outer membrane of mitochondria is mediated by dynamic protein family members called Mfn1 and Mfn2. The fusion between the inner membrane of mitochondria is mediated by a single dynamic protein family protein called fusion protein, optic nerve dystrophy 1 (OPA1). Under specific physiological conditions or under the pressure of harmful external factors, Drp1 aggregates on the surface of the outer membrane of mitochondria, forming a spiral structure to squeeze mitochondria (48). Then, Dyn2 and Drp1 interact cooperatively to regulate the final mitochondrial division step (47). Knockout of Drp1 can inhibit mitochondrial division and enhance mitochondrial networking to form giant mitochondria, which supports the key role of Drp1 in mitochondrial fission (125). Overexpression of Drp1 can accelerate mitochondrial fission and lead to extensive mitochondrial breakage. Division in cells exhausted by Dyn2 can be concluded because the contraction of the mitochondrial membrane fails, resulting in non-division (47). In the brain of rats with chronic fluorosis, the level of mitotic protein increased and the mitochondria of cortical neurons split and redistributed. It has been reported that NaF increased the mRNA and protein levels of Mfn1 and Mfn2 and decreased the mRNA and protein levels of Fis1. At the same time, the level of Drp1 mRNA increased and the protein level of Drp1 reduced. The abnormal mitochondrial division induced by excessive fluoride is characterized by the increase in the number of mitochondria and the structural damage of this organelle. Mitochondrial damage induces the opening of mPTP, and mPTP releases Cyt C from the mitochondria into the cytoplasm (126). The Pro-Caspase 9 is then activated through the intermediate recruitment domain. Once activated, Caspase-9 cleaves and activates the executioner Caspase-3 to induce apoptosis degradation events (127). Fluoride stimulation lead to the increased mRNA expression levels of Cyt C, Caspase-9 and Caspase-3 in a dose-dependent manner, especially in 100 mg/L. Moreover, NaF significantly decreased the level of fission protein and increased the level of fusion protein in the hippocampus of offspring rats, suggesting that fluoride can induce mitochondrial fission / fusion imbalance (49, 50). Once the division / fusion balance is disturbed, the mitochondria will undergo morphological and functional changes. Studies have shown that mitochondrial fission inhibition plays a central role in NaF-induced mitochondrial abnormalities, autophagy defects, increased apoptosis and neuronal damage in human neuroblastoma cells treated with NaF. Mechanically, despite the partial recovery of autophagy, the pharmacological inhibition of mitochondrial fission aggravates NaF-induced mitochondrial defects and cell death by promoting apoptosis. It is suggested that exposure to environment-related levels of fluoride can lead to learning and memory impairment, accompanied by morphological changes of hippocampus mitochondria, such as fission inhibition and accelerated fusion, as well as autophagy deficiency, excessive apoptosis and neuronal loss. The disturbance of the cycle level of identified mitochondrial fission / fusion molecules are closely related to the mental loss of children exposed to fluoride in drinking water for a long time. In general, mitochondrial fission inhibition induces mitochondrial abnormalities, leading to abnormal autophagy and apoptosis, resulting in neuronal death (as shown in Figure 2).

### Fluoride-Induced Mitochondrial Apoptosis Pathway Is Involved in the Potential Molecular Mechanism of Cell Injury Induced by Fluorosis

Fluorosis is primarily due to apoptosis caused by damage to mitochondria. It has been proved that fluoride or fluoride can increase the amount of apoptosis. A large number of literature have reported that mitochondrial-mediated pathway is involved in fluoride-induced apoptosis. As an important subcellular organelle, mitochondria are considered to be the center of the apoptosis pathway. There are three main types of proteins involved in this pathway: Bcl-2 family proteins, caspase and mitochondrial pro-apoptotic proteins. Mitochondrial damage results in the release of Cyt C from mitochondria to cytoplasmic sol, which can activate Caspase-3 and Caspase-9, leading to the formation of apoptosis bodies (46). Cyt C is the key factor that triggers the rapid activation of cysteine and key cellular proteases, which eventually leads to cell death. The release of Cyt C from mitochondria promotes the binding of APAF-1 to Caspase-9, which activates Caspase-9 and initiates the cascade of caspases. In addition, the downstream effector Caspase-3 in the cascade of cysteine aspartate enzymes can be triggered by the activation of Caspase-9, resulting in DNA fragmentation and apoptosis. Apoptosis factors Bcl-2 and Bax play an important role in initiating the permeability of the mitochondrial outer membrane and releasing pro-apoptotic proteins from mitochondria. Bax and Bcl-2 antagonist Bak can help apoptosis through the formation of pure compounds, while Bcl-2 and Bcl-Extra (BclxL) have been reported as endogenous permeability conversion inhibitors (128). In addition, the increase in intracellular reactive oxygen species (ROS), as a signal of oxidative stress, can also activate the apoptosis signal pathway. Once the apoptosis stimulation signal occurs, excessive ROS production contributes to macromolecular oxidation, causing free radicals to attack membrane phospholipids, resulting in membrane damage (129) by inducing lipid peroxidation, mitochondrial membrane depolarization and apoptosis. Sodium fluoride can increase the amount of cAMP and promote apoptosis. Through transcription analysis, mitochondria may play an important role in cAMP-induced apoptosis (130).

### Fluorine-Induced Mitochondrial Autophagy Is Involved in the Potential Molecular Mechanism of Cell Damage Induced by Fluorosis

Autophagy is induced by many cytotoxic stimuli. The relationship between apoptosis and autophagy is complex and difficult to clarify. The process of apoptosis is often accompanied by autophagy (40). Most evidence show that autophagy is the protective mechanism of cell initiation. When autophagy is up-regulated, autophagy can inhibit apoptosis; similarly, apoptosis can also reduce autophagy (131). Under certain conditions, autophagy can not only promote survival, but also promote apoptosis. This process recovers damaged/outdated macromolecules and organelles (132). Autophagy mainly plays a survival role in adapting to unfavorable growth conditions or subsequent cell stress (133). It is mainly involved in differentiation, development, pathogen defense, aging, apoptosis and cell death (134, 135). Autophagy plays a significant role in cell protection under physiological conditions (136). The cytoarchitecture function of autophagy is realized through the negative regulation of apoptosis (137); After fluoride invasion, autophagy and apoptosis are activated at the same time, and can be regulated by the same factors, such as BCL-2 family protein, caspase, ATG protein and p53 (42). On the other hand, autophagy and apoptosis are mutually antagonistic, and autophagy of damaged mitochondrial cells can reduce the transmission of apoptosis signals and protect normal cells. Studies have shown that fluorine can not only induce apoptosis of MC3T3-E1 cells, but also induce autophagy (138). Mammalian mitochondria autophagy is mainly mediated by the following pathways, such as PINK1/Parkin pathway (43), Nix/BNIP3L (42), BNIP3 (44) and FUNDC1 (41). PINK1/Parkin pathway is recognized as one of the important ways of mitochondrial autophagy, which is mainly involved in the elimination of damaged mitochondria (139). PHB2 is an inner mitochondrial membrane protein, which mainly plays a role in regulating the assembly and function of mitochondria. It is the receptor of PINK1/Parkin pathway and a marker of mitochondrial autophagy (140). It is observed that fluorine induced autophagy damage in testis is due to; by inhibiting autophagy degradation and causing autophagy bodies to accumulate in testis. Studies on liver injury caused by dioxapropium poisoning have shown that mitochondria can regulate apoptosis and autophagy, and the damaged mitochondria activate autophagy mechanism to inhibit the release of cytochrome C (Cyt C) and induce apoptosis. Damaged mitochondria can also reduce the accumulation of ROS, thus inhibiting mitochondrial division and preventing its degradation by autophagy (141). Liver damage caused by acute duck poisoning demonstrated that hepatotoxicity on the one hand activated apoptosis and anti-apoptotic system, on the other hand, autophagy protective system was activated, and found that autophagy and apoptotic system inhibited each other, further confirming the mutual inhibitory relationship between autophagy and apoptotic system.

## Conclusion

In this review, we focused on the potential molecular mechanisms of mitochondrial dynamics, energy metabolism, oxidative stress, apoptosis and cell damage induced by steady-state mediated fluorosis. It was found that fluorosis can destroy the ultrastructure of cell mitochondria, break the dynamic balance between fission and fusion, thus causing the transmission of mitochondrial respiratory chain to be blocked, a small amount of electrons escaping from the respiratory chain, and the change of mitochondrial membrane potential, resulting in the increase of ATP, ROS and Ca^2+^ contents, triggering the cascade reaction of capacity, and finally causing cell apoptosis. Mitochondrial dynamics, energy metabolism, oxidative stress, apoptosis and other pathways can be used as molecular targets to explore the probable molecular mechanism of cell damage induced by fluorosis. However, when exploring the cytotoxic mechanism of fluoride from the perspective of mitochondrial dysfunction, it is still found that the specific mechanism of the common regulatory pathway of mitochondrial autophagy and apoptosis after fluorosis cannot be confirmed. In addition, the molecular targets for diagnosis, treatment and intervention measures of fluorosis are still unclear. In mitochondrial dynamics, energy metabolism, oxidative stress, cell apoptosis and mitochondrial homeostasis mediated by calcium ion homeostasis, the interaction between various trade-off mechanisms is not clear, and the precise mechanisms needed to explore further.

## Author Contributions

MW and YY conceived and designed the review. MW and PS analyzed the data. MA, YC, and PS provided manuscript editing. All authors statistically analyzed, discussed, critically revised the contents, and approved the final manuscript.

## Funding

This work was supported by the major science and technology projects of Tibet autonomous region (XZ202101ZD0005N), the key R&D plan of Bayi District, Nyingchi City (2021-GX-SY-01), and the basic research funds of China Agricultural University (2021TC002).

## Conflict of Interest

The authors declare that the research was conducted in the absence of any commercial or financial relationships that could be construed as a potential conflict of interest.

## Publisher's Note

All claims expressed in this article are solely those of the authors and do not necessarily represent those of their affiliated organizations, or those of the publisher, the editors and the reviewers. Any product that may be evaluated in this article, or claim that may be made by its manufacturer, is not guaranteed or endorsed by the publisher.
